# Viruses Occur Incorporated in Biogenic High-Mg Calcite from Hypersaline Microbial Mats

**DOI:** 10.1371/journal.pone.0130552

**Published:** 2015-06-26

**Authors:** Rutger De Wit, Pascale Gautret, Yvan Bettarel, Cécile Roques, Christian Marlière, Michel Ramonda, Thuy Nguyen Thanh, Huy Tran Quang, Thierry Bouvier

**Affiliations:** 1 Centre for Marine Biodiversity, Exploitation and Conservation (MARBEC),Université de Montpellier, CNRS, IRD, Ifremer, Place Eugène Bataillon, Case 093, 34095, Montpellier, France; 2 Université d’Orléans, ISTO, UMR 7327, 45071, Orléans, France and CNRS, ISTO, UMR 7327, 45071 Orléans, France and BRGM, ISTO, UMR 7327, BP 36009, 45060, Orléans, France; 3 Institut des Sciences Moléculaires d'Orsay (ISMO), Université Paris-Sud, CNRS, Bâtiment 350, Université Paris-Sud, 91405, Orsay Cedex, France; 4 DRED Services Communs de la Recherche/ Centre Technologique de Montpellier, Université de Montpellier, 34095, Montpellier, France; 5 Nanobiomedicine group, Laboratory Ultrastructure, Department of Virology, National Institute of Hygiene and Epidemiology (NIHE), 1 Yersin Street, Hai Ba Trung, 1000, Hanoi, Vietnam; Auckland University of Technology, NEW ZEALAND

## Abstract

Using three different microscopy techniques (epifluorescence, electronic and atomic force microscopy), we showed that high-Mg calcite grains in calcifying microbial mats from the hypersaline lake “La Salada de Chiprana”, Spain, contain viruses with a diameter of 50–80 nm. Energy-dispersive X-ray spectrometer analysis revealed that they contain nitrogen and phosphorus in a molar ratio of ~9, which is typical for viruses. Nucleic acid staining revealed that they contain DNA or RNA. As characteristic for hypersaline environments, the concentrations of free and attached viruses were high (>10^10^ viruses per g of mat). In addition, we showed that acid treatment (dissolution of calcite) resulted in release of viruses into suspension and estimated that there were ~15 × 10^9^ viruses per g of calcite. We suggest that virus-mineral interactions are one of the possible ways for the formation of nano-sized structures often described as “nanobacteria” and that viruses may play a role in initiating calcification.

## Introduction

Extremely small bacteria (0.02–0.1 µm size), and very small bacteria (0.1–0.3 µm), commonly referred to as “nanobacteria” [1] have been invoked as initiating agents for biogenic calcification in benthic systems [1–6]. Nanoscale bodies in different sedimentary settings and calcium carbonate-rich rocks [1, 2, 7], in deep Earth sand stones [8] as well as in a Martian meteorite [9] have been interpreted as putative nanobacteria. In medicine, they have been referred to play a key role in calcification related diseases [10–12], where calcium oxalate and calcium phosphate precipitation occurs as an apparent self-replicating process. The enigmatic putative nanobacteria were defined as “extremely small cellular forms, widespread in nature and closely associated with the formation of inorganic precipitates and geological strata” [13]. However, the cellular and prokaryotic nature of these nanometer-scale spherical and ovoid bodies has been challenged both by geologists, microbiologists and human health scientists [14].

Cisar et al. [15] failed to detect nucleic acids and have suggested that the self-replicating particles are complexes of minerals and organic macromolecules. A more recent study also failed to detect nucleic acids and showed that the addition of DNAase and RNAase did neither result in arrest of the self-replication process nor induce a change of morphology. In contrast, the particles contained fetuin, an antimineralisation protein, which seemed to be involved in inducing the self-propagation of the calcium hydroxyapatites mineral complexes [16]. The authors claimed that the existence of nanobacteria can now definitively be ruled out and suggested to name the self-propagating mineral complexes “nanons” [16]. In addition, another study showed that X-ray spectroscopic signatures of self-replicating calcified nanoparticles cultured from human samples were similar to calcified proteins and clearly different from that of calcified bacteria [17].

In conclusion, there is no clear evidence that the putative nanobacteria of 20–100 nm sizes [1, 10] are indeed *bona fide* prokaryotes, which comprise sufficient DNA, RNA, ribosomes, membranes and metabolic machinery to ensure their independent replication. Different theoretical calculations of the minimum size of a prokaryote converge towards a minimum volume of 0.014–0.06 µm^3^ with a diameter ranging from 140–300 nm [18–21]. Non-living nanobacteria-resembling structures have been reported to include calcite crystals surrounded by an amorphous carbonate layer [22], proteinaceous spheroids produced during enzyme-mediated tissue degradation [23], CaCO_3_ crystals prepared in human blood under in vitro conditions [24], and nano-scale particles or “nanoballs” in cultures of sulfate-reducing bacteria. The latter represent early stages of carbonate precipitation and either developed on the cell surface [25] or were excreted within extracellular polymeric substances [26]. Objects resembling “nanobacteria” have also been experimentally reproduced by abiotic calcite precipitation from solutions containing dissolved organic matter or bacterial fragments including lysates that contained bacterial phages [27]. New insights in crystallisation have highlighted that biomineralisation is often based on so-called “non-classical crystallisation” [28] showing oriented crystal growth at surfaces, which may include sheaths, cell walls and outer membranes of micro-organisms as well as mineral surfaces coated by organic matter. The nanocrystals thus formed can be assembled into larger units as mesocrystals by a process called mesoscopic transformation [28]. However, the mesoscopic transformation not only leads to a single crystal with complex morphologies, but also to nanoparticles embedded in an organic matrix [28]. Such a mechanism may explain the observations of i) calcite with a granular structure consisting of coalescing nanocrystals [29], ii) the nanometer-scale mixture of calcium carbonate crystals and organic molecules in recent microbialites [30] and iii) the occurrence of spherulites in experimental tufas [31].

While viruses have been neglected until recent times, we consider that these particles should be studied for their possible role in the formation of nano-scale mineral structures or nanoparticles included in crystals. Sizes of marine viruses generally range from 20 to 300 nm with many examples around 40–80 nm [32]. Very efficient nucleic acid binding fluorescent dyes have been instrumental in the discovery that viruses are the most abundant living particles in the ocean and freshwater environments [33, 34]. Virus concentrations in the water column typically range from 10^4^ to 10^8^ per mL and the specific viruses attacking bacteria (virulent bacteriophages) exert a strong control of bacterial populations, which results in bacterial loss rates that are comparable to the loss rates caused by grazing [35]. Recent reports also clearly show that viruses are also ubiquitous and abundant in sediments and biofilms [36–39], with densities as high as 10^8^ to 1.5 × 10^10^ particles per cm^3^, and in microbial mats and stromatolites [40–42]. Incubation experiments of diluted samples and measurements of incorporation of radiolabelled substrates in viral genomes indicate that virus production rates are high in sediments, implying that lytic viruses exert a strong control on the dynamics of benthic prokaryotes [37]. Nevertheless, it is surprising that generally very few benthic bacterial cells showed visible signs of viral infection [39, 43].

Viruses are composed of a protein capsule with the nucleic acid in its interior. Since the capsule can interact directly with the cations and anions in solution [44] and thus potentially influence the precipitation processes, we hypothesised that the “nanobacteria” like particles embedded in calcium carbonate micro grains could have been confounded with viruses. Recent studies have investigated viruses for their capacities to promote mineral precipitation and, consequently, how their mineral encasement may represent a fossilisation mechanism. Thus, interactions between different viruses and iron have been studied under experimental conditions [44] and in the natural acidic environment of Rio Tinto [45]. Silicification was also explored experimentally for hot spring silicifying conditions, including the short-term and reversible fossilisation of bacteriophage T4 and the *Sulfolobus* spindle-shaped virus Kamchatka [46, 47] and the long-term experimental fossilisation of viruses hosted by hyperthermophilic Archaea [48]. Observations in hot spring biofilms suggest that also in natural environments viruses can be preserved (i.e., formation of silicified nanoparticles inside the extracellular polymeric substances) [49]. A recent study showed that amorphous Mg-Si precipitated at the surface of viral particles in hypersaline microbial mats and that experimental simulation of diagenesis resulted in replacement of the Mg-Si by Mg carbonate [42]. The field of astrobiology has also neglected the study of viruses for a long time. However, there is nowadays a growing focus on viruses, virus-constituents and fossilised viruses as biomarkers in the search for past or present extra-terrestrial life [50], particularly on the planet Mars and the Jupiter II Europa satellite [51].

Hence, we envisaged that the “nanobacteria” like particles embedded in calcium carbonate micro grains could be viruses. Choosing a calcifying microbial mat [52, 53] for this study, we aimed (i) to detect and quantify free and attached viruses in different layers of these mats and (ii) to explore whether viruses do occur within the fine grain biogenic calcium carbonate.

## Materials and Methods

### Ethics statement

Lake "La Salada de Chiprana" (Aragón, NW Spain) is a Ramsar wetland site (since 1994), a Site of Community Interest according the Habitats Directive in the European Union (SCI since 1997) and has been declared a natural reserve since 2006 by the Gobierno de Aragón. Permission for Field Studies in Chiprana lake has been granted by the Departamento de Agricultura, Ganadería y Medio Ambiente of the Gobierno de Aragón (Zaragoza, Spain).

### Study site and sampling

Calcifying microbial mats built by the cyanobacterium *Coleofasciculus chthonoplastes* (Thuret ex Gomont) M. Siegesmund, J. R. Johansen & T. Friedl, formerly known as *Microcoleus chthonoplastes* [54] and filamentous *Chloroflexus*-like bacteria (*CLB*) were sampled in the hypersaline lake “La Salada de Chiprana” in NE Spain (41°14’30”N, 0°10’50”W) during March and September 2007. Its general limnology [55] and paleolimnology [56] have been described in details. The wax and wane of the *C*. *chthonoplastes* mats during the last two decades have been related to the concomitant variations of the lake level and its water column salinities [57]. The microbial structure of multilayered microbial mats built by *C*. *chthonoplastes* and *CLB* reflecting several years of growth has been described in detail by [52]. The mats sampled in 2007 for this study corresponded to young mats that recolonised the sediments after the demise of the mats during the period 2002–2006, which was related to excessive growth of the foxtail stonewart *Lamprothamnium papulosum* var. *papulosum* f. aragonense (Prósper) and a dystrophic crises in 2006 [57]. Twenty cm long, 10 cm wide and 5 cm deep portions of the mat and underlying sediment were sampled under ~0.5 m water depth and stored in separate plastic trays filled with the Chiprana lake water to be transported to the laboratory.

Vertical cross-sections of the sediment with the microbial mats were placed along a ruler and photographed with a digital camera with a macro-objective to measure the average depth horizon of the distinct coloured laminations of the mats. Five to six different horizontal layers of the mats were sampled using scalpels and razorblades. Small aliquots of the different samples were observed with phase-contrast and epifluorescence microscopy to identify diatoms, cyanobacteria and the *CLB* (see [52] for microscopic criteria). The different depth layers were used for studying the concentrations of (i) free viruses occurring in suspension in the interstitial water or in an aqueous phase that occurs soaked into the extracellular organic matter, (ii) attached viruses adsorbed on sediment particles and on the organic matrix (iii) those that can be liberated by dissolving the CaCO_3_ minerals by acidification (see below). For the mats sampled in September 2007 we also separated and purified the fine grained biogenic CaCO_3_ minerals from the five different layers.

### Counting of virus particles in different sediment fractions

Liquid fractions were collected from the five microbial mat layers by centrifugation of 10 g of sample. The supernatant fractions comprise the interstitial pore water and probably also water that is soaked into the polymer matrix. The numbers of viruses were counted in these liquid fractions. Therefore, before virus counting, 500 µL of these supernatant solutions were fixed with formaldehyde (2% final concentration) for two hours in the dark and diluted twice in tris(hydroxymethyl)aminomethane (TRIS, 10 mM) buffer with

Ethylenediaminetetraacetic acid (EDTA, 1 M) at pH = 7.8 (TE buffer).

Attached viruses were separated from the organic matter and mineral particles by the following procedure: An aliquot of ~100 mg of the homogenised dried pellet was weighted to determine its dry weight with a precision of ± 1 mg, and suspended in 3.9 mL of a pyrophosphate solution (10 mM). This suspension was incubated in the dark for 20 minutes at 4°C and subsequently sonicated three times in a UltraSonic NEY bath (1 min, 72 kHz, 70 µm amplitude) before virus counting.

On several occasions we treated the pellets with acid to study whether viruses were liberated by dissolution of biogenic (Ca,Mg)CO_3_ crystals. Therefore, we compared a non-acidified control with the acid treatment. Two aliquots of approximately 100 mg of the homogenised dried pellet were weighted, the control aliquot was suspended in 0.95 mL of MilliQ water and the aliquot for the acid treatment was incubated with 0.95 mL of an aqueous solution of HCl (0.1 M). After exactly 10 min the acid incubation was stopped by the addition of 0.95 mL of a solution of NaHCO_3_ (0.1 M) resulting in a pH of 7 to 7.5. In parallel 0.95 mL of MilliQ water was added to the control. Two mL of a pyrophosphate solution (20 mM) were added to both tubes, which were further processed as above. We also considered that the acid treatment itself could result in the destruction of viruses. Therefore, we also acidified the extracted liquid fractions (porewater and water loosely bound to extracellular polymers, see above), which virtually did not contain (Ca,Mg)CO_3_ grains, according the same procedure (0.1 mL incubated with 0.95 mL HCl (0.1 M) during 10 min; and subsequently neutralised with 0.95 mL of a solution of NaHCO_3_ (0.1 M)). The counting of these acidified samples was compared with that of the control.

Viruses were counted by epifluorescence microscopy following a staining with fluorescent dye [58]. Solutions with suspended viruses from field samples and the different treatments abovementioned were filtrated onto 0.02-µm-pore-size Al_2_O_3_ Anodisc filter (Whatman Inc.) and stained. Briefly, the 0.02 µm filters were prewetted on the filtration support with the TE buffer solution (10 mM Tris, 1M EDTA, pH = 7.8). A portion from the different extractions described above was homogenously spread on the filter and the liquid was sucked trough the filter using a depression pressure of 20 kPa. The dry filters were deposited on 30 µL of an aqueous SYBR Gold nucleic acid stain solution (2.5 µL of 10,000 × stock solution in 1 mL autoclaved MilliQ water) for 15 min in the dark at ambient temperature. Filters were then remounted onto the filtration set, rinsed three times with TE buffer and stored at -20°C until microscopic observation. The stained Anodisc filters were mounted on a glass slide with a drop of anti-fading solution (1 g Redoxon and 0.1 g Vitamin E in filtered isotonic PBS water). SYBR Gold is a dye that specifically stains nucleic acids DNA and RNA, and the nucleic acid SYBR Gold complexes show a strong yellow-green coloured fluorescence under blue light excitation [58]. Microscopic observations were conducted with an Olympus epifluorescence microscope (AX70) using a blue broad-band filter for excitation (488 nm). Viruses were <0.2 µm in diameter, intensely yellow-green coloured and could be distinguished from bacteria by their form and size (the latter are >0.5 µm rods or coccoids). At least 400 stained viruses were counted per sample. The analytical error related to the preparation and filtering of the sample and microscopic counting was σ < 5%.

### Extraction and analysis of biogenic carbonate grains

(Ca,Mg)CO_3_ grains were purified by destruction of the organic matter in which they were embedded in the mat. Therefore, an aliquot of the solid part of the different sediment layers was treated with a solution of 3.5% sodium hypochlorite to liquefy the organic matter. The aliquot was put into a 50 mL Falcon centrifuge tube and 40 mL of a 3.5% sodium hypochlorite solution in MilliQ water was added. The tubes were incubated on an orbital shaker for 20 h and afterwards spun down by centrifugation during 10 min at 100 g (700 RPM rotor, r = 18 cm). Mineral particles including the biogenic (Ca,Mg)CO_3_ grains sedimented in the pellet. The microscopic observations confirmed that the supernatant virtually did not contain (Ca,Mg)CO_3_ minerals in suspension. The supernatant was discarded and the pellet was resuspended in 40 mL of MilliQ water and incubated on an orbital shaker for two hours to dissolve the liquefied organic matter. This preparation was again centrifuged during 10 min at 100 g (700 RPM rotor, r = 18 cm) and the supernatant was again discarded. This procedure was repeated until phase contrast microscopy showed that all remains of solid organic matter had been dissolved. The pellet was dried overnight at 37°C and subsequently sieved through a 70 µm sieve. Although we do not know the efficiency of this method in terms of recovery, fine white powder was obtained that was enriched in biogenic (Ca,Mg)CO_3_ mineral grains.

A weighted portion of each powder sample was used to count viruses both for control and acid treatment conditions as described above. Other portions of these powder samples were embedded in epoxy resin. Sections of 70 nm in thickness were cut using an ultra-microtome (Leica, EM UC6). Several sections were deposited onto 200 mesh, formvar-coated copper grids for transmission electron microscopy (TEM) analysis and other sections were deposited on microscope glass slides for scanning electron microscopy (SEM), atomic force microscopy (AFM), and subsequently for epifluorescence microscopy.

The carbonate grains present in the sections have been characterised by SEM linked with energy dispersive X-ray spectrometry (SEM-EDS) and by AFM, in order to study the features indicative for their authigenicity and biogenicity. Elemental maps were acquired with a resolution of 512 × 384 pixels during 15 to 30 minutes. Multiple point analyses were performed on selected calcite grains, during 300 sec (10 to 15 point analyses in area of 20 to 60 µm width, each of them with an analytical error for weight % of σ < 1%). Calcium and magnesium were assumed fully associated with CO_3_^2-^ to form (Ca,Mg)CO_3_ (Mg-calcite). High-resolution images were acquired with a Hitachi S4500 SEM high-resolution field emission instrument operating at 1 kV and with an AFM D3100 from Bruker Metrology Inc., Santa Barbara, CA. The latter apparatus combines optical microscopy and atomic force microscopy. AFM was operated in intermittent contact mode; i.e., the tip is oscillated at constant frequency (close to the resonance frequency). The amplitude of vibration is kept constant to a set value by using a feedback loop controlling the distance between the tip and the surface by varying the voltage of the vertical piezo actuator. These variations of voltage are used to build the height image. Phase angle between the excitation signal of the cantilever and the detected signal when the sample surface is scanned and represented as phase images, as this has the potential to reveal contrast on heterogeneous materials. AFM observations were performed in ambient conditions at a relative humidity of 50 ± 5% and a temperature of 22 ± 2°C.

### Microscopy of virus and bacteria-like particles in (Ca,Mg)CO_3_ grains

TEM was used to detect viruses and bacteria included into the calcium carbonate grains. The sections of biogenic (Ca,Mg)CO_3_ grains were deposited onto carbon-coated copper grids, stained with aqueous uranyl acetate (20 g L^-1^) for 60 s, and then washed twice with distilled water for 20 s. Although uranyl acetate can potentially cause damages to the smallest calcium carbonate bodies by causing partial dissolution, this compound can still be used for obtaining TEM images of the organic matrix enclosed inside small calcified bodies [59, 60]. Hence, inclusions of viruses were observed in TEM-mode with a Hitachi, S-4800 STEM equipped with an energy-dispersive X-ray spectrometer (XEDS) (Horiba, E-max) and analytical software. For the XEDS analysis, the STEM was used in TEM mode, operated at 15 kV with a high probe current, a working distance of 15 mm and a total accumulation time for each X-ray spectrum of 45 seconds (live time). For the selected areas we optimized the contrast and brightness to obtain the best resolution of the bacterial and viral shapes. The contours of these particles were drawn manually and their forms were submitted to particle analysis (length, with and area) and X-ray microanalysis. To quantify the contribution of the supporting film, we recorded the spectrum of a particle-free area of identical size and shape adjacent to that particle, and subtracted it from the main particle spectrum. The elemental composition parameters of bacteria and viruses were recorded for 13 replicates each. The N signal has been deconvoluated from the O and C peaks by the INCA version 5.05 (Oxford Instruments, Gometz la Ville, France) software optimized for the study of living material. For statistical comparisons within and between these two groups we used the logarithm of the elemental ratios (N/P) as recommended for compositional data [61], which also results in scale invariance, i.e., the magnitude of the signal is now independent of the choice for numerator and denominator, because log(N/P) = -log(P/N). Elements are also expressed in weight % considering a contribution from hydrogen assumed to be 1/6 (wt/wt) that of carbon. Data in weight % were transformed in Mol kg^-1^ as follow: Mol kg^-1^ = (weight % / molecular mass of the element) × 100.

AFM was used to study the topography, surface and subsurface characteristics of the calcium carbonate grains to screen for possible viruses inclusions. AFM observations were performed in intermittent contact mode on a D3100 from Bruker Metrology Inc., Santa Barbara, CA allowing to obtain height and phase images (see above). The phase angle value as suggested is directly related to the dissipated energy per oscillation cycle [62]. Viscoelasticity of the material as well as its adhesion properties may be involved in this dissipation. Hence, the phase signal is sensitive to hard particles lying up to 80 nm under the surface of a softer material [63].

For the observation of viruses embraced into calcium carbonate grains, 20 µL of the SYBR Gold solution were added on top of the embedded slices of carbonate grains mounted on glass slides. This solution was allowed to react during 15 min at ambient temperature. The SYBR Gold in excess was removed by gently rinsing the slides with TE buffer and subsequently added the antifading. Stained viruses and bacteria were observed and enumerated as described above.

## Results

### Quantification of virus-like particles in the microbial mat layers

**Fig 1:**
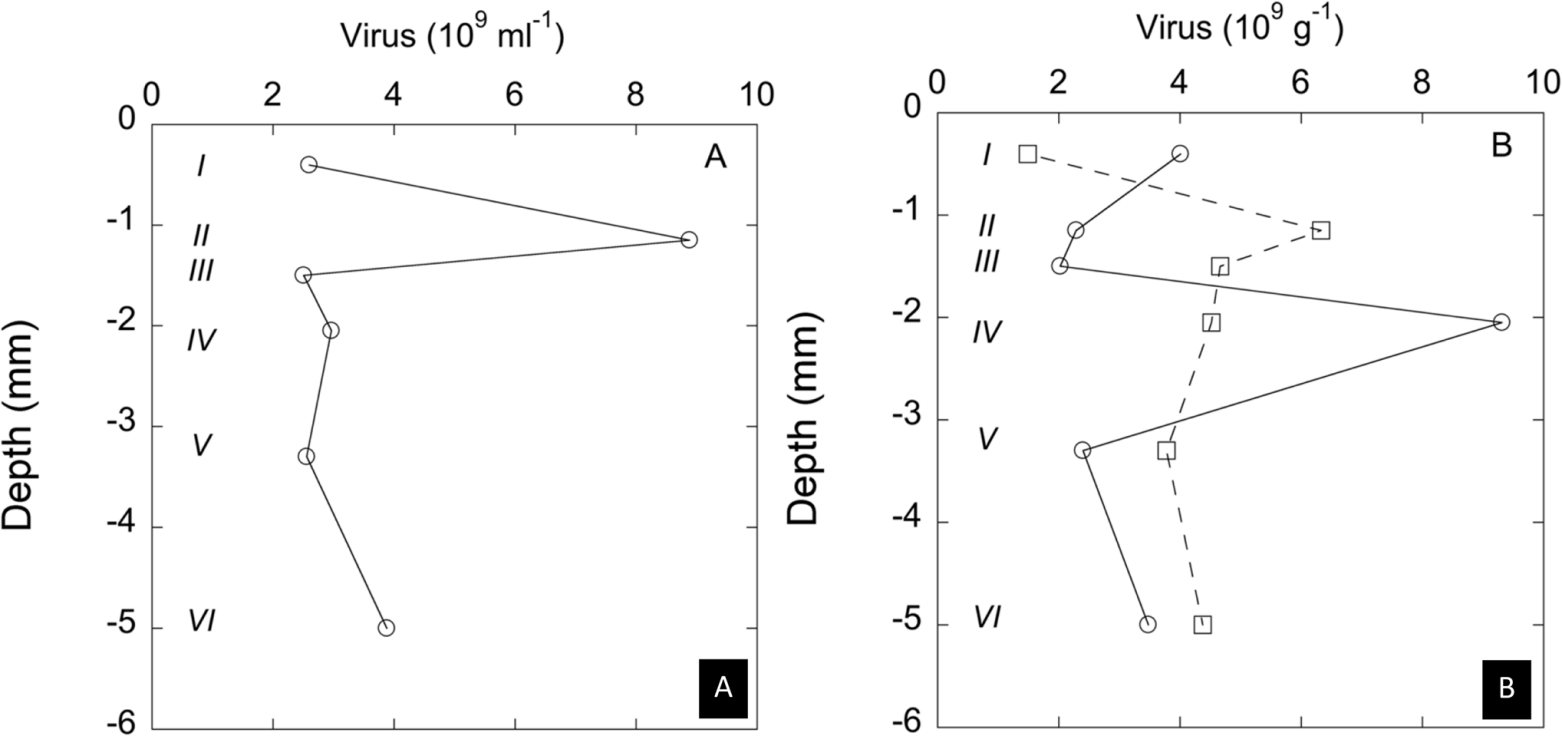
Virus observed in different depth layers (see below) and different fractions of the microbial mat dominated by diatoms, *Coleofasciculus chthonoplastes*, and *Chloroflexus*-like bacteria (CLB), sampled in March 2007. Left panel (A): Virus counts in the extracted water fraction (pore water and water soaked into the extracellular polymer matrix). Right panel (B): Virus attached to solid organic and mineral matter. (circles = without acidification, squares = after 10 min of acidification, see Methods). Description of the different layers: I: the top layer from 0 to 0.8 ± 0.2 mm depth, comprised dense populations of diatoms of the genera: *Frustula*, *Cymbella*, *Denticula*, *Nitzschia* and few bundles of *C*. *chthonoplastes* and filaments of CLB; II: layer from 0.8 ± 0.2 mm to 1.5 ± 0.2 mm depth that separated very well from the top layer and comprised lesser densities of diatoms with high quantities biogenic high-Mg calcite grains (cf. Fig 3); III: locally a very fine layer was observed at 1.5 ± 0.3 mm depth that was particularly enriched in biogenic high-Mg calcite embedded in an organic matrix; IV: A layer located below the high densities of biogenic calcium carbonate crystals occurred layer B from 1.5 ± 0.3 mm to 2.6 ± 0.3 mm depth which comprising bundles of *C*. *chthonoplastes* and filaments of CLB; V: layer from 2.6 ± 0.3 mm to 4 ± 0.3 mm depth corresponded to a transition zone where large amounts of mineral particles sand grains and biogenic calcite occurred intertwined with of *C*. *chthonoplastes* and filaments of CLB; VI: layer comprising black coloured sediment ranging from 4 ± 0.3 mm to 6 ± 0.5 mm depth.

The mat sampled in March 2007 corresponded to a very young microbial mat (less than two years, see Methods). Fig 1 describes depth profiles of viruses in the mat and the legend describes the composition of the different layers based on morphological light microscopy observations. The concentrations of the viruses suspended in extracted water fraction ranged between 2 and 10 × 10^9^ viruses per mL and the values for the different layers are depicted in Fig 1A. Using these samples for testing the effect of the acidification procedure (10 min in 0.1 M HCl, see Methods), it was found that this treatment induced losses of on average 60% of free viruses (N = 10). Fig 1B shows the concentrations of viruses attached to the solid organic matter and mineral particles, which ranged from 2 to 9.5 × 10^9^ per g dry weight observed in layers III and IV, respectively. This figure also shows the concentrations found after the 10 min acidification treatment. In the layers I and IV, which were dominated by diatom populations and by communities of *C*. *chthonoplastes* associated with *CLB*, respectively, the number of viruses was 40% lower than in the non-acidified control. This can be explained to a large extent by losses induced by acidification. In contrast, the layers II, III, V and VI showed significantly higher viruses numbers after acidification, which shows that viruses were liberated by dissolution of the (Ca,Mg)CO_3_ grains. The layers II and III, which were particularly rich in biogenic (Ca,Mg)CO_3_ grains, showed a 4.5 and 3.3 fold increase upon acidification. This indicates that a large part of the viruses associated with organic and mineral solids was actually included in the biogenic (Ca,Mg)CO_3_ grains.

**Fig 2:**
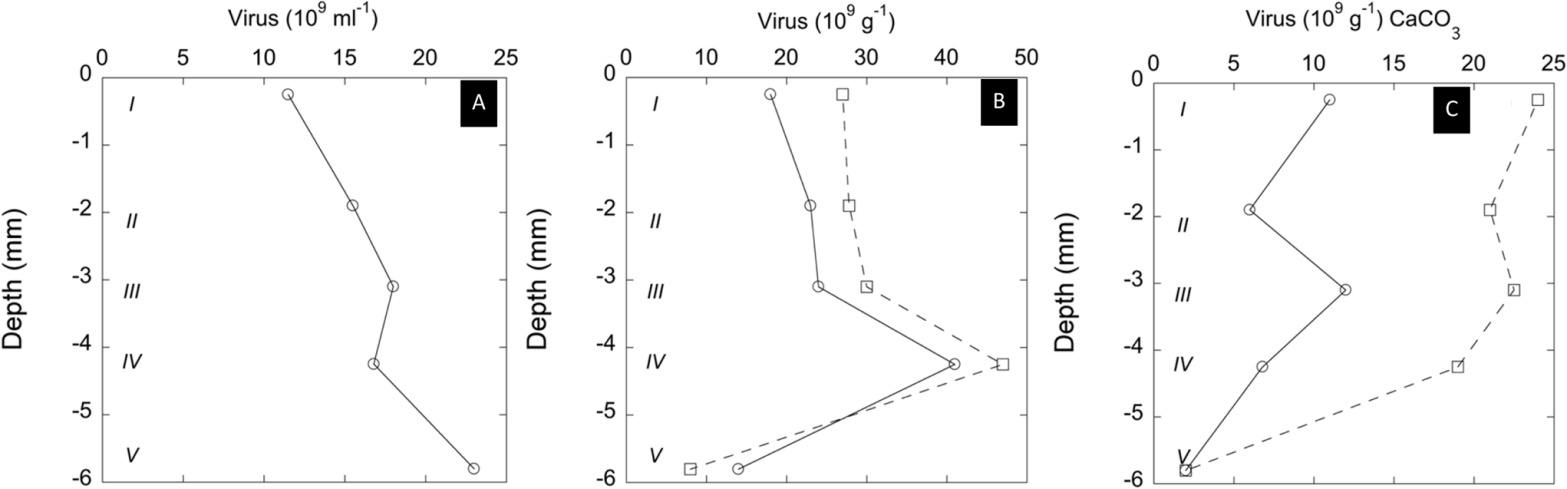
Virus observed in different depth layers (see below) and different fractions of the microbial mat dominated by diatoms, *Coleofasciculus chthonoplastes*, and *Chloroflexus*-like bacteria (CLB), sampled in September 2007. A: Virus counts in the extracted water fraction (pore water and water soaked into the extracellular polymer matrix). B: Virus attached to solid organic and mineral matter. (circles = without acidification, squares = after 10 min of acidification, see Methods). C: Virus associated with extracted and purified carbonate grains observed on the outside of the carbonate grains (circles) and after 10 min of acidification (squares). See Methods for details. Description of the different layers: I: the yellow brown toplayer (0–1 ± 0.1 mm depth) was dominated by the diatom species belonging to the genera *Frustula*, *Cymbella*, *Denticula*, *Nitzschia*; II: layer B (1 ± 0.1 mm to 2.3 ± 0.2 mm depth) comprised bundles of *C*. *chthonoplastes* and filaments of CLB; III: layer (2.3 ± 0.2 mm to 3.9 ± 0.2 mm depth) corresponding to a transition zone with *C*. *chthonoplastes* and filaments of CLB, some of them showing signs of degradation; IV: deeply black coloured sediment (3.9 ± 0.3 mm to 5 ± 0.3 mm depth); V: layer (5 ± 0.3 mm to 6.4 ± 0.4 mm depth) was grey coloured with a lot of sand grains, other mineral particles. Biogenic high-Mg calcite grains were observed by microscopy in layers I, II, III, and IV.

The mat sampled in September 2007 represented a slightly more developed mat of *C*. *chthonoplastes* and *CLB* together with diatoms. The top layer I was dominated by diatoms and the layer II by communities of *C*. *chthonoplastes* associated with *CLB* on top of older degrading mat (III) and sediment (IV and V). In general, both the concentrations of freely suspended viruses in the extracted water and the viruses attached to the solid organic matter and mineral particles were higher in September than in March (cf. Fig 1A and 1B with 2A and 2B). In the four top layers (I–IV), where we observed biogenic (Ca,Mg)CO_3_ minerals in high amounts, the acidification resulted in increased viruses counts ranging from 51 to 12%, observed in layers I and IV, respectively (see Fig 2B). Again this increase is attributed to the liberation of viruses included in the carbonate grains. Only in layer V, we observed a decrease of 37% of viruses that can be explained to a large extent to losses induced by acidification.

The biogenic carbonate minerals extracted and purified from the different layers represented a white powder comprising cauliflower-like agglomerations of very small globules (see description below). We observed that viruses still occurred at high densities in these preparations on the outside of these biogenic carbonate agglomerations, despite the vigorous treatment with hypochlorite and multiple washings with MilliQ water. The acidification systematically resulted in an increase of 1.1 to 1.5 × 10^10^ viruses per g of carbonate grain for layers A to D, and only in layer E we did not observe any effect of acidification (see Fig 2C).

### Microscopy of purified biogenic carbonate aggregates

**Fig 3:**
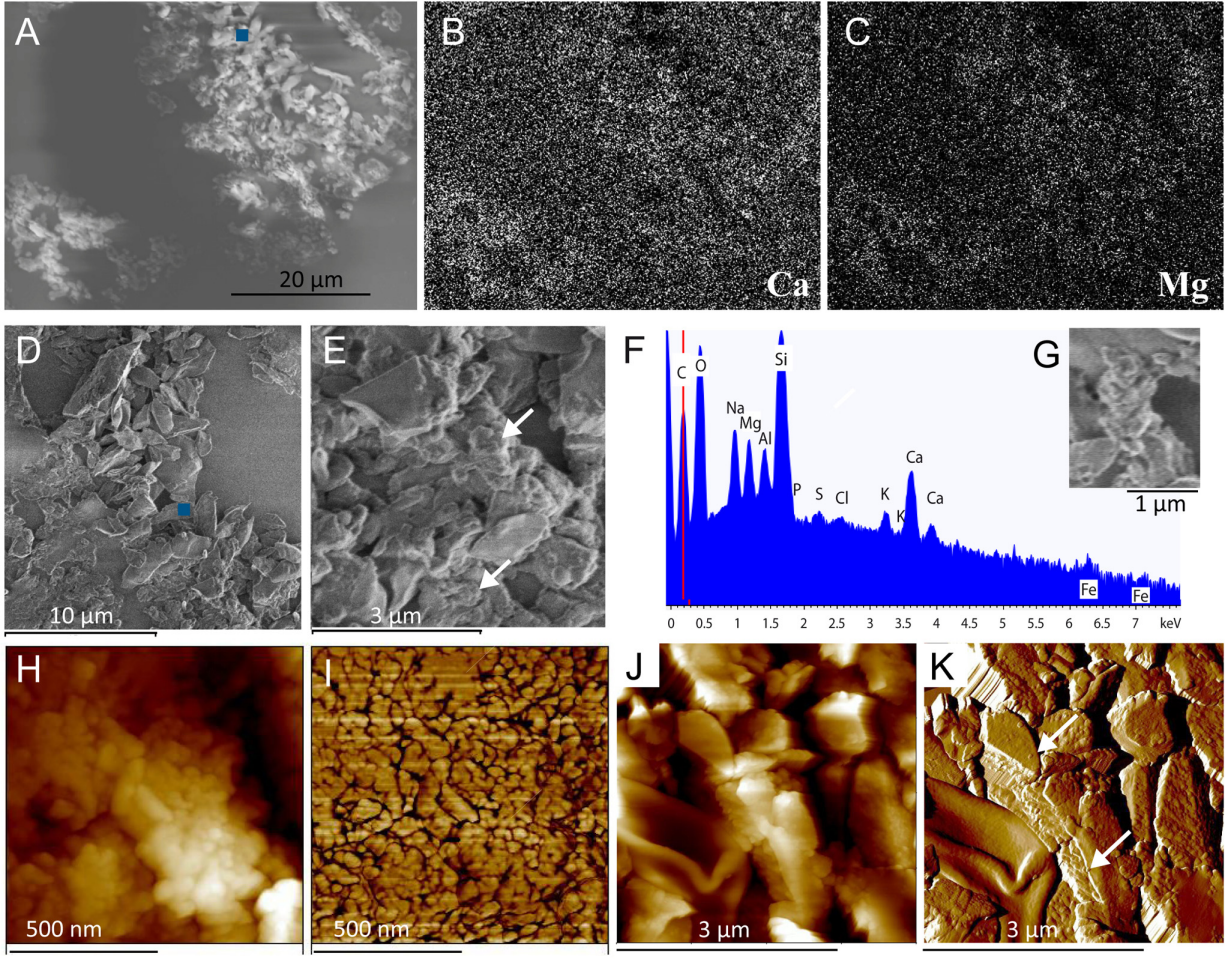
Biogenic calcite, extracted from the microbial mats, observed in scanning electron microscopy linked with energy dispersive X-ray spectrometry and in atomic force microscopy. A: SEM picture of a cross section of carbonate grains showing a surface area of about 50 × 40 µm cutting through two agglomerates of very small globules and large crystals. B and C: Maps of calcium (B) and magnesium (C) obtained by EDS analysis in the same area. D-E: High-resolution images showing the variety in size and shape of mineral grains and crystals that compose biogenic calcium carbonate agglutinates. The arrows point towards smaller agglomerates of roundish micro-grains. F: EDS spectrum of the (Ca,Mg)CO_3_ grain for the location indicated by the blue dots in A and D (note that the high Si peak, due to the glass slide, masks the Si present in the clay colloids which also comprise Al and K). G: Details of an agglomerate of roundish micro-grains showing micromorphologies of very small rounded globules. H, I: AFM (in intermittent contact mode) images of a 1 × 1 µm surface area showing the globular structure of the individual (Ca,Mg)CO_3_ micro-grains and presented as height (H) and phase (I) images. J, K: AFM images of a 4 × 4 µm surface area in the cross section in AFM showing topography (J) and phase (K) images at a magnification comparable to that used for SEM. White arrows point to viruses that have been studied at higher magnification, i.e., the viruses illustrated in Fig 4D–4E.

The authigenic and biogenic characters of carbonate agglutinates were assessed by morphological and morphometric features of individual composing globules, as well as geochemical composition (Mg calcite). Fig 3 shows a typical example of the observations of the carbonate agglutinates made with SEM linked with energy dispersive X-ray spectrometry and AFM. At lower magnification (Fig 3A), the carbonate grain gives the impression of cauliflower agglutinates in the range from 5 to 50 µm. At higher magnification (D, E) 3–5 µm-size crystals are found to co-occur with agglomerates of roundish structures (white arrows in E). The latter appear to consist of very small rounded globules (G for SEM, and H,I for AFM images). The agglomerates of very small rounded globules are identified as high-Mg calcite, i.e., 25–35 mol % Mg^2+^ in the calcite grains (average 29.4 ± 2.9 mol % Mg^2+^; N = 23). The large crystals are less abundant and consist of 4–10 mol % Mg^2+^-calcite (average 6.8 ± 1.6 mol % Mg^2+^; N = 10).

**Fig 4:**
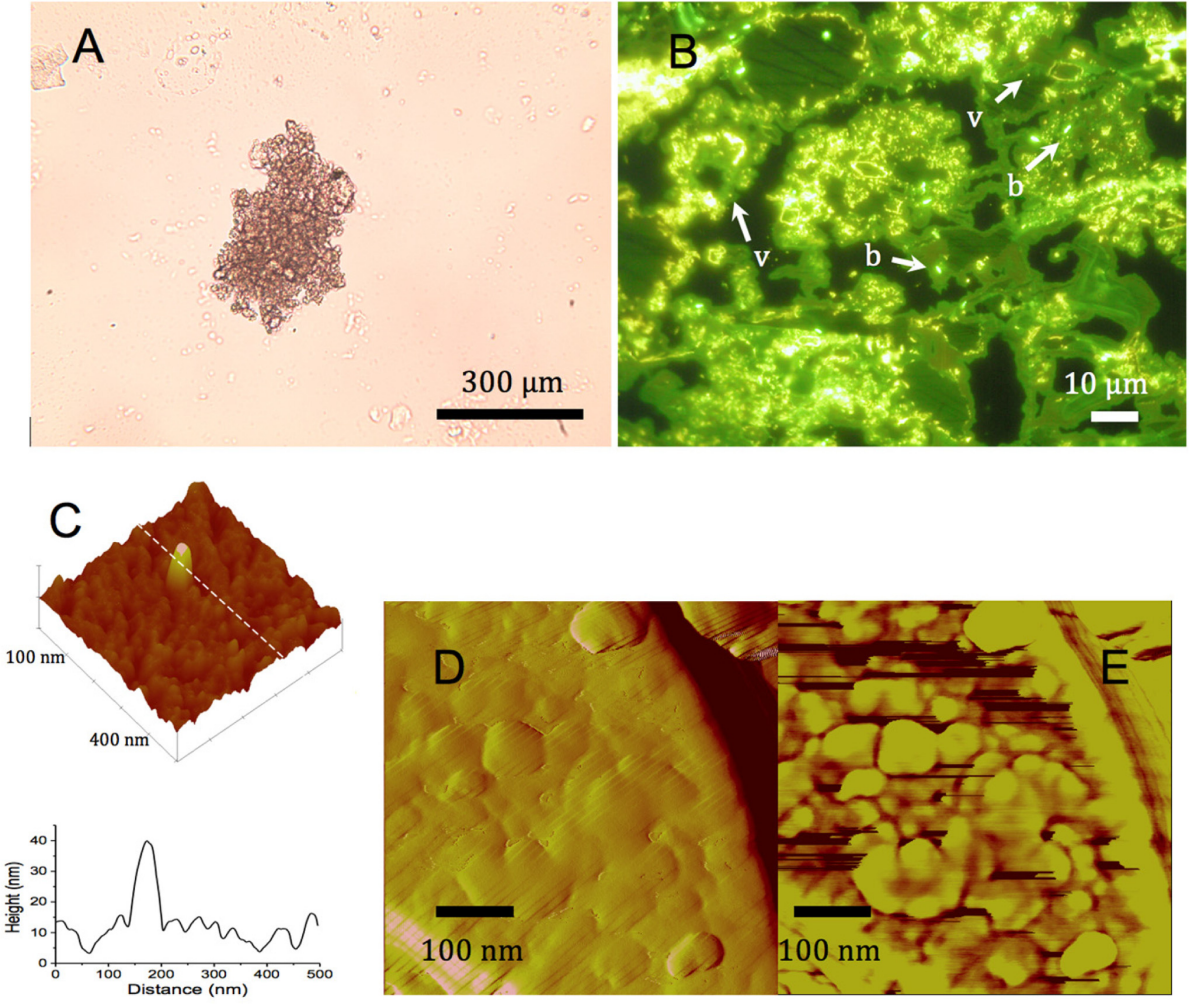
A: Biogenic high-Mg calcite minerals observed under light microscopy (phase contrast). B: Light micrograph of the carbonate crystal section stained with the nucleic acid dye SYBR Gold showing specific yellow-green fluorescence of bacteria (b) and viruses (v). C: three-dimensional presentation of an AFM image of a section of a biogenic calcium carbonate mineral showing a topographic anomaly caused by an included particle emerging 30–40 nm above the crystal surface interpreted as a viruses, the panel also includes a topographic presentation along the broken white line. D: AFM height image of a 500 × 500 nm surface area of a biogenic calcium carbonate grain. E: same area as in panel D but according a phase image representation.

Fig 4A shows a phase contrast light microscopy image of the cauliflower-like agglomeration of fine micritic high-Mg calcite. Fig 4B shows a SYBR Gold staining of the thin sections, showing high numbers of very small and intensely fluorescent dots and occasionally a larger (0.3–1 µm length) rod or coccoid form with a lesser fluorescent intensity. The small particles are very similar to viruses observed with the same method in other aquatic systems both in water column and sediment compartments [58]. Viral counts of 10 biogenic carbonate grains (agglutinates) ranged from 2.3 to 8.2 × 10^4^ virus per grain with an average of 4.6 × 10^4^ virus per grain. Considering that crystal grain volume was 1.1 × 10^−7^ cm^3^ (N = 30) and assuming that their density was equal to 2.7 as for an abiotic calcite crystal [64], we estimated that there were approximately 15 × 10^9^ virus per g of calcite.

AFM observations in intermittent contact mode are depicted in Fig 4C and 4D and 4E. The surface of the carbonate grains in the thin sections showed the frequent occurrence of regularly shaped anomalies emerging from the crystal surface as that depicted in Fig 4C (three-dimensional height image); the section along transect (along the white dashed line in Fig 4C) reveals (bottom of Fig 4C) that this specific structure mounted 30 to 40 nm above the crystal surface. Many similar anomalies with the same dimensional characteristics were observed regularly distributed over the crystal surface. Hence, the morphometrics indicate that a body of 40–80 nm is incorporated in the crystal at its very surface (Fig 4C). Fig 4D and 4E show the height and phase image respectively for another 500 × 500 nm sample surface. This area did not show the same spectacular anomalies mounting to more than 30 nm above the surface as those of Fig 4C, although less pronounced topology suggest that nanoparticles are included in the crystal and only slightly emerge above the surface (Fig 4D). However, the phase image (Fig 4E) reveals additional structures, which can be interpreted as below surface structures. It has been reported that mapping the phase angle in “tapping-mode” AFM, i.e., AFM operated in intermittent contact mode, allows revealing structures below the surface. The penetration depth of these phase-image observations depends on the hardness of the surface and has been reported to range from 20 to 80 nm below surface for hard [65] and soft surface [63], respectively.

**Fig 5:**
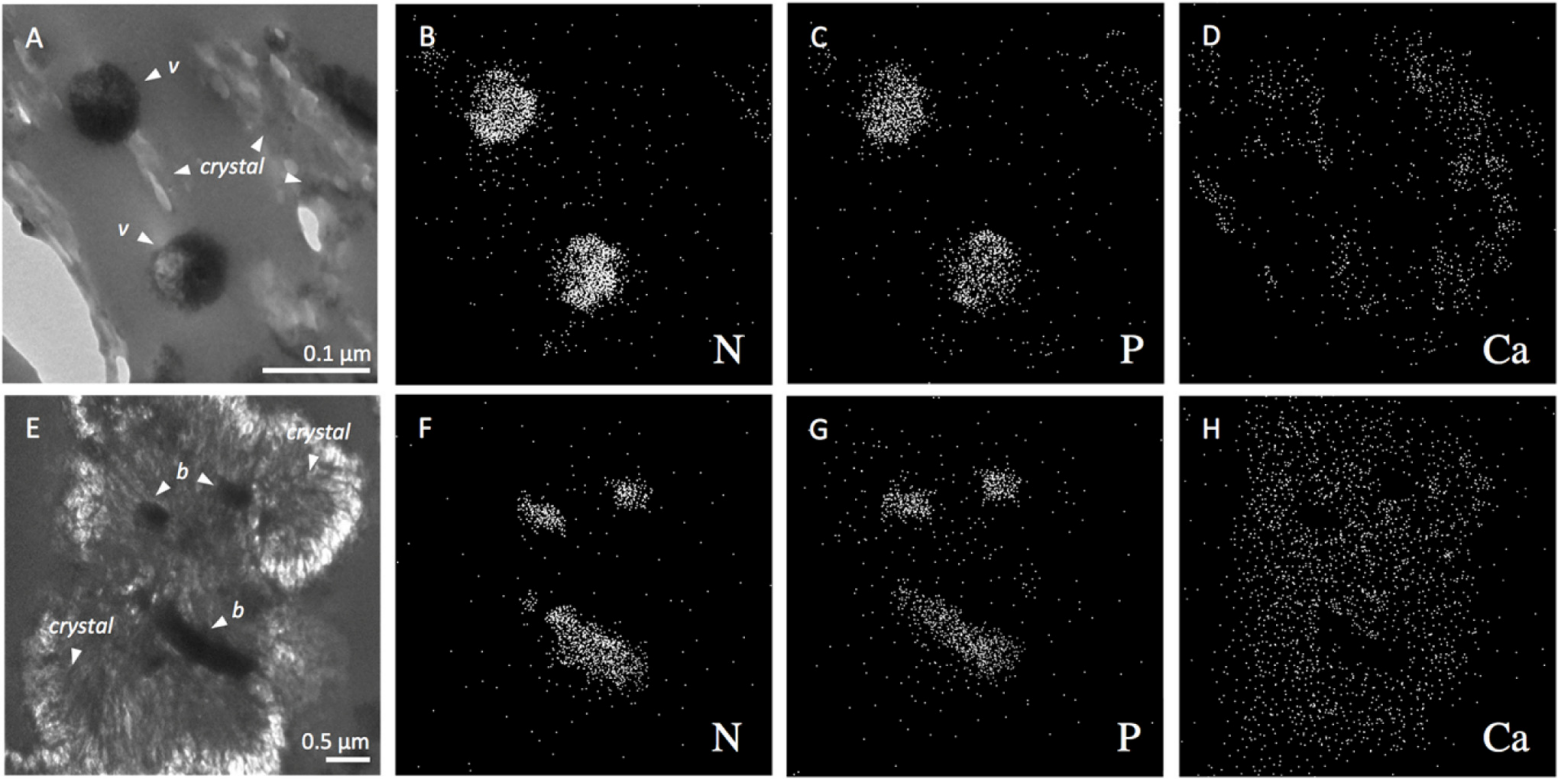
TEM image of cross-sections of high-Mg calcite showing apparent polyhedral-like viruses marked v (A) and bacteria marked b (E), and their elemental composition shown for the same fields in (B, C, D) and (F, G, H), respectively. The XEDS maps of nitrogen (B, F), phosphorus (C, G), and calcium (D, H), respectively, showing that viruses and bacteria contain nitrogen and phosphorus, with calcium mainly located in the mineral part of the high-Mg calcite grain (Ca,Mg)CO_3_.

**Fig 6:**
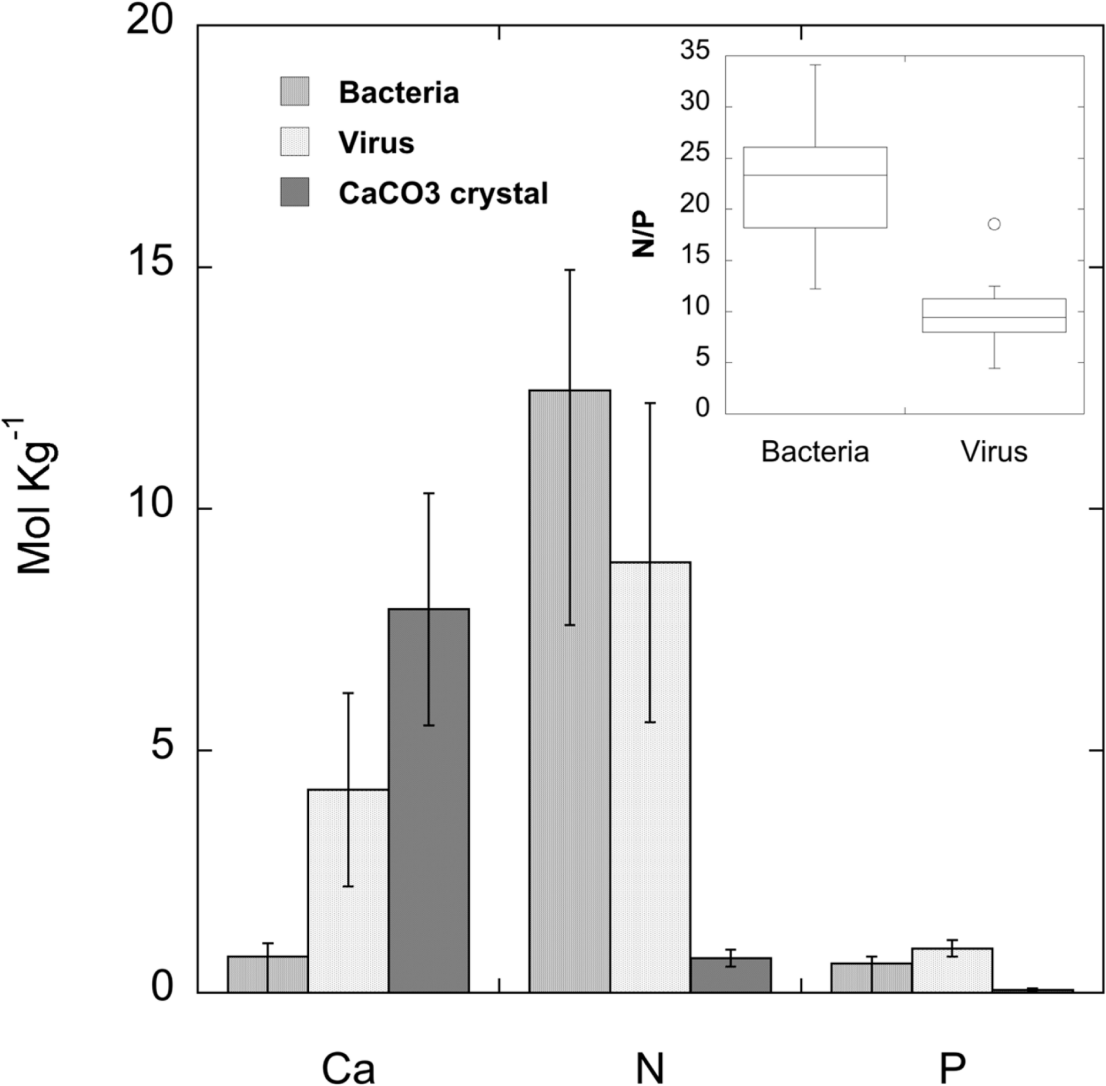
Calcium, nitrogen and phosphorus content obtained by X-ray analysis of single bacterial cells and viruses incorporated in the biogenic calcium carbonate crystal, and of the crystal itself. **N = 13.** Inlet: Box and whisker plot of N/P ratio in bacteria and virus.

Fig 5A shows the TEM image of two apparently polyhedral-like viruses imbedded in the high-Mg calcite. Their capsid sizes ranged from 50 to 80 nm (N = 50). Less frequently we also observed bacterial cells incorporated in the mineral grains (Fig 5E). XEDS analysis of viruses and bacteria showed that all of them contained nitrogen and phosphorus (Fig 5B, 5C, 5F, 5G and Fig 6). A one-sided student T comparison of log-converted molar N/P ratios showed highly significant differences (p = 9.70 × 10^−7^) between bacteria (geometric mean = 21.0, median = 23.3 mol N / mol P) and viruses (geometric mean = 9.25, median = 9.45 mol N / mol P), respectively. In contrast, the mineral high-Mg calcite had very low content of nitrogen and phosphorus.

## Discussion

We showed high densities of viruses in both fractions of the microbial mat, i.e., (i) in the extracted water, indicating their prevalence in the pore water or in the water matrix soaked into the organic matrix, and (ii) attached to the organic and mineral solid matrix. By SYBR Gold staining of their nucleic acids, these small particles are visualised as fluorescent dots that morphometrically resemble the viruses observed in other aquatic systems both in the water column and sediment compartments [33, 58]. It may appear surprising that the stained viral particles (also called virus-like particles), which are often much smaller than 0.4 µm the resolving power of visible light, can be observed by fluorescence microscopy. This is due to the high fluorescence intensity of the particles that thus appear larger than their actual size [58].

The observed high abundance in this hypersaline microbial is coherent with other findings in various types of hypersaline waters such as crystallizer ponds [66, 67] and hypersaline lakes where viral densities can reach up to 10^9^ viruses mL^-1^ [42, 68–71]. Their high stability and specific ability to persist and proliferate in these apparently restrictive habitats have been proposed to partly explain their remarkable abundance [72].

In addition, we showed that in layers of the mat that are particularly rich in biogenic carbonate grains, an acid treatment resulted in significant increases of the number of viruses obtained in suspension. This increase reflects the liberation of viruses by dissolution of the biogenic calcium carbonate. However, this increase probably underestimates the amount of viruses included in the biogenic calcium carbonate grains, since the acid treatment itself may result in a loss of 60% of viruses as we observed for the water-extracted samples.

Within the mat, the biogenic calcium carbonate grains occur as conglomerates, which show a cauliflower-like morphology under SEM at lower magnification. These comprise agglomerates of very small globules (0.5–1 µm), which contained up to 35 mol % Mg^2+^, and larger particles of Mg calcite. The latter appear as partially geometric crystals albeit irregularly shaped (i.e., subeuhedral crystals), which contained less Mg than the former agglomerates. The agglutinates also contain low amounts of sulfur due the associated organic matrix, as well as traces of Fe and other elements (for example K, Al) of entrapped clay colloids (Fig 3F). Such morphological and geochemical characters strongly suggest that these agglutinates have formed *in situ* within the microbial mat [73–76] and are not derived from an allochthonous source. The high magnesium content of the calcite is most likely related to the high molar Mg^2+^/Ca^2+^ ratio in the water of the lake, which was 21.4 ± 10.4 during the last two decades [57]. We extracted and purified these high-Mg calcite grains from the mat sampled in September 2007 and showed that in several layers acid treatment resulted in liberation of 1.1 to 1.5 × 10^10^ viruses per g of high-Mg calcite.

In addition to SEM observations of whole grains, we also prepared thin sections of these mineral grains for epifluorescence microscopy, AFM and TEM analyses to visualize the occurrence of the viruses within the mineral grains. The SYBR Gold staining and characteristic fluorescence under blue light excitation showed that the particles contained nucleic acids, i.e., either RNA or DNA (Fig 4B). Counting the number of viruses confirmed that their densities were above 1.5 × 10^10^ viruses per g of high-Mg calcite. However, epifluorence microscope images, as shown in Fig 4B, do not allow measuring the sizes of the viruses, since the virus particles appear larger than their actual size [58] due to their high fluorescence intensity. Our AFM observations showed topological anomalies at the surface of the crystal as nano-scale bodies that emerged 30 to 40 nm above the crystal surface (Fig 4C). The phase image (Fig 4E) reveals that bodies of these sizes also occurred in the crystal below its surface. These anomalies in the biogenic high-Mg calcite complexes with a very regular morphology and size, about 30–80 nm, have been interpreted as the signatures of virus capsids, either partly embedded and emerging from the crystal surface (Fig 4C) or occurring fully embraced slightly below the surface (Fig 4D and 4E). Indeed, these very small bodies cannot be confounded with other calcite-related organominerals of similar sizes because the contrasts revealed by the phase signal in AFM indicates the presence of heterogeneous materials, in the terms of viscoelasticity and adhesion properties, within the crystal. The image observed in the phase image (Fig 4E) is strikingly similar to AFM images obtained for Pox viruses [77]. The TEM examination of thin sections of the calcium carbonate granules revealed the apparent polyhedral-like morphology of these viruses with their capsid size ranging from 50 to 80 nm. We should consider that the viral particles may have suffered from some degree of deformation during their incorporation into the carbonate grains and that we were not able to reveal the detail that can be obtained for the viral particles obtained from the lysis of the host in a bacteriophage host culture. TEM images of comparable quality for viruses extracted from microbial mats [42] have been interpreted as icosahedral-like morphologies, which represent a specific case of a polyhedral capsid, i.e., a polyhedral with 20 faces.

The sizes of the viruses in the high-Mg calcite grains fit within the range measured in a recent meta-analysis of marine viruses (ranging from 25 to 187 nm, with a mean of 56 ± 13 nm, N = 2600 [78]). Such morphotypes of relatively small sizes may indicate that viruses in the microbial mats could be either bacteriophages or small viruses of eukaryotes. Indeed, the high occurrence of cyanobacteria (*C*. *chthonoplastes*) and *Chloroflexus*-like bacteria (CLB) in the mats may naturally justify the presence of phages of the *Caudovirales* order, whose capsid is typically icosahedral with a diameter comprised between 50 and 90 nm [72, 78, 79]. The tails may have been lost during their incorporation in the crystal. Alternatively, a part of the incorporated viruses could be also the results of diatoms infections since these microalgae were also abundant in the mats. Previous reports have shown that viruses of diatoms are usually small (i.e., <70 nm) [80–83] to access the inner cell through the pores of the siliceous frustule. Interestingly diatom pore sizes are mostly comprised between 40 and 200 nm [84], which is compatible with our observations of small sized viruses.

Bacterial morphotypes, generally with a size of 200 to 300 nm width, and up to 1500 nm length (see Fig 5E), were much less frequent than viruses. The XEDS images of cross-sections of the high-Mg calcite showed that while the grain morphology was reflected by the Ca map, low values of Ca were observed where the virus and bacterial particles were located in the grains. The virus and bacterial particles were characterised by high N and high P, although the molar ratios were very different between both groups of particles. Recently, the C, N, P elemental composition of viruses has been calculated for a number of well-studied viruses and a general biophysical scaling model has been proposed to predict C:N:P ratios as a function of virus size, based on protein and nucleic acids contents [85]. Hence it has been predicted that the N:P molar ratio of viruses may range from 5 to 15 for 100 nm and 20 nm capsids, respectively. Fifty percent of the viruses incorporated in the grains and observed by XEDS showed a N:P molar ratio between 7.9 and 11.3 with a geometric mean value of 9.25. In contrast, the larger inclusion particles identified as bacteria had higher C/N molar ratios with a geometric mean of 21.0. This value is higher than the typical Redfield ratio, which is frequent for bacteria in freshwater lakes [86]. The presence of phosphorus was also detected in experimentally silicified viruses [46], which according these authors and in agreement with [85] must be attributed to the genomic DNA in the capsid, since the bacteriophage T4 they studied does not possess an envelope and its proteins are not known to be phosphorylated.

Viruses are composed of a protein capsule with the nucleic acid in its interior, and the tail and tail fibers also consist of protein. Different amino acids polymerised in the proteins, and particularly the bicarboxylic acids, may induce biomineralisation. Pacton et al. have shown that amorphous Mg-Si precipitated at the surface of viral particles in hypersaline microbial mats and that diagenesis, particularly in anoxic environments, can result in replacement of the Mg-Si by Mg carbonate [42]. Our study shows that viruses are incorporated in high-Mg calcite crystals that are formed in the actively photosynthesizing mat [53]. Collectively, both studies show that viruses may play a role in biogenic carbonate precipitation. This may by either via an indirect route, involving silicified viruses as an intermediate phase during diagenesis [42] or through direct incorporation of viruses into growing high-Mg calcite crystals [this study]. For the *Coleofasciculus*/*CLB* mats in this study it has been clearly shown that calcification is driven by photosynthesis [53], which acts during daytime as the intrinsic alkalinity engine [87]. According this model, the pH shift induced by photosynthesis increases the proportion of the carbonate ion, which increases the ion product (Ca^2+^, CO_3_^2-^) and thus helps to overcome the kinetic barrier for the calcium carbonate precipitation. However, phospholipid or lipid-protein complexes can initiate mineralisation process [88]; in general, biomineralisation may be initiated by biological macromolecules [15, 89, 28]. Most likely such a biomineralisation process involves “non-classical” crystallisation *sensu* [28], which may give rise to irregularly shaped metacrystals of calcite interspersed with nano-scale bodies that could have been misinterpreted as nanobacteria in some cases. Hence, biogeochemical control of calcification has been shown to be responsible for the morphologies of the micro grains obtained [90–92]. Viruses can potentially play similar roles. We therefore suggest that the alkalinity engine related to the photosynthetic activity of the mats could act in synergy with viruses; the latter probably functioning as crystallisation nuclei or as templates for oriented crystal growth.

Alternatively, it can be envisioned that the viruses are haphazardly incorporated in the growing calcite crystals. A haphazard inclusion can be favoured due to the very high abundance of the viruses in the mat and it is expected that a growing high-Mg calcite grain has a very high probability of encountering a virus during its growth. Upon encounter it is thus possible that the growing crystal simply engulfs the virus rather than pushing it away. However, also the inclusion of viruses in the calcite can potentially influence the mineral precipitation process, e.g., by biogeochemically driving the morphologies of the high Mg-calcite grains.

The observation of viruses included in biogenic calcite minerals offers very interesting avenues for research in geobiology, ecology and evolution. First of all, it shows that virus mineral interactions may give rise to nanoparticles that could have been misinterpreted as “nanobacteria” in some cases. Secondly, this mechanism could also result in the fossilisation of viruses. The long-term conservation of viral material in the geological record will allow the study of the viral genome and proteome in old strata.

## References

[1] Folk RL. SEM imaging of bacteria and nannobacteria in carbonate sediments and rocks. J Sediment Res 1993; 63: 990–999.

[2] Folk RL. Nannobacteria and the precipitation of carbonate in unusual environments. Sediment Geol 1999;126: 47–55.

[3] Pedone VA, Folk RL. Formation of aragonite cement by nanobacteria in the Great Salt Lake, Utah. Geology 1996;24: 763–765.

[4] Vasconcelos C, McKenzie JA. Microbial mediation of modern dolomite precipitation and diagenesis under anoxic conditions (Lagoa Vermelha, Rio de Janeiro, Brazil). J Sediment Res 1997;67: 378–390.

[5] Camoin G, Gautret P, Montaggioni LF, Cabioch G. Nature and environmental significance of microbialites in Quaternary reefs: the Tahiti paradox. Sediment Geol 1999;126: 271–304.

[6] Sprachta S, Camoin G, Golubic S, Le Campion T. Microbialites in a modern lagoonal environment: nature and distribution (Tikehau atoll, French Polynesia). Palaeogeogr Palaeocl 2001;175: 103–124.

[7] Russo F, Gautret P, Mastandrea A, Perri E. Syndepositional cements associated with nannofossils in the Marmolada Massif: evidences of microbially mediated primary marine cements? (Middle Triassic, Dolomites, Italy). Sediment Geol 2006; 185: 267–275.

[8] Uwins PJR, Webb RI, Taylor AP. Novel nano-organisms from Australian sandstones. Am Mineral 1998;83: 1541–1550.

[9] McKay DS, Gibson EK, Thomas-Keprta KL, Vali H, Romanek CS, Clemett SJ et al. Search for past life on Mars: Possible relic biogenic activity in Martian meteorite ALH84001. Science 1996;273: 924–930. 8688069 10.1126/science.273.5277.924

[10] Kajander EO, Çiftçioglu0020N. Nanobacteria: an alternative mechanism for pathogenic intra- and extracellular calcification and stone formation. P Natl Acad Sci USA 1998;95: 8274–8279. 9653177 10.1073/pnas.95.14.8274 PMC20966

[11] Folk RL. Nannobacteria in the natural environment and in medicine. Alpe Adria Microbiology Journal 1998;7: 87–95.

[12] Çiftçioglu N, Bjöklund M, Kuorikoski K, Bergström K, Kajander EO. Nanobacteria: an infectious cause for kidney stone formation. Kidney Int 1999;56: 1893–1898. 10571799 10.1046/j.1523-1755.1999.00755.x

[13] Hamilton A. Nanobacteria: gold mine or minefield of intellectual enquiry? Microbiology today 2000;27: 182–184.

[14] Urbano P, Urbano F. Nanobacteria: Facts or Fancies? PLoS Pathog 2007; 3(5): e55 10.1371/journal.ppat.0030055 17530922 PMC1876495

[15] Cisar JO, Xu DQ, Thompson J, Swaim W, Hu L, Kopecko DJ. An alternative interpretation of nanobacteria-induced biomineralization. P Natl Acad Sci USA 2000;97: 11511–11515. 11027350 10.1073/pnas.97.21.11511 PMC17231

[16] Raoult D, Drancourt M, Azza S, Nappez C, Guieu R, Rolain JM et al. Nanobacteria are mineralo fetuin complexes. PLoS Pathog 2008 2 8; 4(2):e41 10.1371/journal.ppat.0040041 18282102 PMC2242841

[17] Benzerara K, Miller VM, Barell G, Kumar V, Miot J, Brown GE, Lieske JC. Search for microbial signatures within human and microbial calcifications using soft X-ray spectromicroscopy. J Investig Med 2006; 54: 367–379. 10.2310/6650.2006.06016 17169258

[18] Koch AL. Microbial physiology and ecology of slow growth. Microbiol Mol Biol 1997;R 61: 305–318. 9293184 10.1128/mmbr.61.3.305-318.1997 PMC232613

[19] Maniloff J. Nannobacteria: Size limits and evidence. Science 1997;276: 1776–1776 9206833 10.1126/science.276.5320.1773e

[20] Psenner R, Loferer M. Nannobacteria: Size limits and evidence. Science 1997;276: 1776–1777. 9206835

[21] Velimirov B. Nanobacteria, ultramicrobacteria and starvation forms: a search for the smallest metabolising bacterium. Microbes Environ 2001;16: 67–77.

[22] Benzerara K, Menguy N, Guyot F, Dominic D, Gillet P. Nannobacteria-like calcites at surface of the Tataouine meteorite. P Natl Acad Sci USA 2003;100: 7438–7442. 12792020 10.1073/pnas.0832464100 PMC164604

[23] Schieber J, Arnott HJ. Nannobacteria as a by-product of enzyme-driven tissue decay. Geology 2003;31: 717–720.

[24] Martel J, Young YD-E. Purported nanobacteria in human blood as calcium carbonate nanoparticles. P Natl Acad Sci USA 2008;105: 5549–5554, 10.1073/pnas.0711744105 18385376 PMC2291092

[25] Aloisi G, Gloter A, Krüger M, Wallmann K, Guyot F, Zuddas P. Nucleation of calcium carbonate on bacterial nanoglobules. Geology 2006;34: 1017–1020.

[26] Bontognali TRR, Vasconcelos C, Warthmann RJ, Dupraz C, Bernasconi1 SM, McKenzie JA. Microbes produce nanobacteria-like structures, avoiding cell entombment. Geology 2008;36: 663–666.

[27] Kirkland BL, Lynch FL, Rahnis MA, Folk RL, Molineux IJ, McLean RJC. Alternative origins for nannobacteria-like objects in calcite. Geology 1999;27: 347–350.

[28] Niederberger M, Cölfen H. Oriented attachment and mesocrystals: Non-classical crystallization mechanisms based on nanoparticle assembly. Phys Chem Chem Phy 2006;8: 3271–3287. 10.1039/B604589H 16835675

[29] Perri E, Tucker M, Mawson M. Biotic and abiotic processes in the formation and diagenesis of Permian dolomitic stromatolites (Zechstein Group, NE England). J Sediment Res 2013;83: 896–914. 10.2110/jsr.2013.65

[30] Benzerara K, Menguy N, López-García P, Yoon TH, Kazmierczak J, Tyliszczak T et al Nanoscale detection of organic signatures in carbonate microbialites. P Natl Acad Sci USA 2006;103: 9440–9445. 16772379 10.1073/pnas.0603255103 PMC1480426

[31] Pedley M, Regerson M, Middleton R. Freshwater calcite precipitates from in vitro mesocosm flume experiments: a case for biomediation of tufas. Sedimentology 2009;56: 511–527. 10.1111/j.1365-3091.2008.00983.x

[32] Weinbauer M. Ecology of prokaryotic viruses. FEMS Microbiol Rev 2004;28: 127–181. 15109783 10.1016/j.femsre.2003.08.001

[33] Fuhrman JA. Marine viruses: biogeochemical and ecological effects. Nature 1999;399: 541–548. 10376593 10.1038/21119

[34] Suttle CA. Viruses in the sea. Nature 2005;437: 356–361. 16163346 10.1038/nature04160

[35] Fuhrman JA, Noble RT. Viruses and protists cause similar bacterial mortality in coastal seawater. Limnol Oceanogr 1995;40:1236–1242.

[36] Danovaro R, Dell’Anno, Trucco A, Serresi M, Vanucci S. Determination of virus abundance in marine sediments. Appl Environ Microbiol 2001;67: 1384–1387. 11229937 10.1128/AEM.67.3.1384-1387.2001 PMC92740

[37] Danovaro R, Dell’Anno A, Corinaldesi C, Magagnini M, Noble R, Tamburini C. et al. Major viral impact on the functioning of benthic deep-sea ecosystems. Nature 2008;454: 1084–1087. 10.1038/nature07268 18756250

[38] Glud RN, Middelboe M. Virus and bacteria dynamics of a coastal sediment: implication for benthic carbon cycling. Limnol Oceanogr 2004;49: 2073–2081.

[39] Filippini M, Buesing N, Bettarel Y, Sime-Ngando T, Gessner MO. Infection paradox: high abundance but low impact of freshwater benthic viruses. Appl Environ Microbiol 2006;78: 4893–4898. 10.1128/AEM.00319-06 PMC1489317 16820485

[40] Rice G, Stedman K, Snyder J, Wiedenheft B, Willits D, Brumfield S et al Viruses from extreme thermal environments. P Natl Acad Sci USA 2001;98: 13341–13345. 11606757 10.1073/pnas.231170198 PMC60872

[41] Desnues C, Rodriguez-Brito B, Rayhawk S, Kelley S, Tran T, Haynes M. et al Biodiversity and biogeography of phages in modern stromatolites and thrombolites. Nature 2008;452: 340–343. 10.1038/nature06735 18311127

[42] Pacton M, Wacey D, Corinaldesi C, Tangherlini M, Kilburn M, Gorin GE et al Viruses as new agents of organomineralization in the geological record. Nature communications 2014;5:4298, 10.1038/ncomms5298 24989676

[43] Bettarel Y, Bouvy M, Dumont C, Sime-Ngando T. Virus-Bacterium Interactions in Water and Sediment of West African Inland Aquatic Systems. Appl Environ Microbiol 2006;72: 5274–5282. 16885276 10.1128/AEM.00863-06 PMC1538746

[44] Daughney ChJ, Châtellier X, Chan A, Kenward P, Fortin D, Suttle CA et al. Adsorption and precipitation of iron from seawater on a marine bacteriophage (PWH3A-P1). Mar Chem 2004;91: 101–115.

[45] Kyle JE, Pedersen K, Ferris FG. Virus mineralization at low pH in the Rio Tinto, Spain. Geomicrobiol J 2008;7(8): 338–345.

[46] Laidler JR, Stedman KM. Virus silicification under simulated hot spring conditions. Astrobiology 2010;10: 569–576. 10.1089/ast.2010.0463 20735248

[47] Laidler JR, Shugart JA, Cady SL, Bahjat KS, Stedman KM. Reversible inactivation and dessication tolerance of silicified viruses. J Virol 2013;87: 1327–1329. 10.1128/JVI.02825-13 PMC3838281 24109222

[48] Orange F, Chabin A, Gorlas A, Lucas-Staat S, Geslin C, Le Romancer M et al. Experimental fossilisation of viruses from extremophilic Archaea. Biogeosciences 2011;8: 1465–1475.

[49] Peng X, Xu H, Jones B, Chen S, Zhou H. Silicified virus-like nanoparticles in an extreme thermal environment: implications for the preservation of viruses in the geological record. Geobiology 2013;11: 511–526. 10.1111/gbi.12052 24102946

[50] Griffin W. The quest for extraterrestral life: what about viruses? Astrobiology 2013;13: 774–783. 10.1089/ast.2012.0959 23944293

[51] Stedmann K, Blumberg BS. The NASA Astrobiology institute virus focus group workshop and field trip to Mono and Mammoth lakes, CA, June 22–24, 2004. Astrobiology 2005;4: 441–443. 16078864 10.1089/ast.2005.5.441

[52] Jonkers HM, Ludwig R, De Wit R, Pringault O, Muyzer G, Niemann H et al. Structural and functional analysis of a microbial mat ecosystem from a unique hypersaline inland lake: “La Salada de Chiprana” (N.E. Spain). FEMS Microbiol Ecol 2003;44: 175–189. 10.1016/S0168-6496(02)00464-6 19719635

[53] Ludwig R, Al-Horani F, de Beer D, Jonkers HM. Photosynthesis-controlled calcification in a hypersaline microbial mat. Limnol Oceanogr 2005;50: 1836–1843.

[54] Siegesmund MA, Johansen JR, Karsten U, Friedl T. *Coleofasciculus* gen. nov. (Cyanobacteria): morphological and molecular criteria for revision of the genus *Microcoleus* Gomont. J Phycol 2008;44: 1572–1585. 27039870 10.1111/j.1529-8817.2008.00604.x

[55] Vidondo B, Martinez B, Montes C, Guerrero M-C. Physicochemical characteristics of a permanent Spanish hypersaline lake: la Salada de Chiprana (NE Spain). Hydrobiologia 1993;267: 113–125.

[56] Valero-Garces BL, Navas A, Machin J, Stevenson T, Davis B. Responses of a saline lake ecosystem in a semiarid region to irrigation and climate variability. The history of Salada Chiprana, central Ebro basin, Spain. Ambio 2000;29: 344–350.

[57] De Wit R, Guerrero MC, Legaz A, Jonkers HM, Blocier L, Gumiaux C. et al. Conservation of a permanent hypersaline lake: management options evaluated from decadal variability of *Coleofasciculus chthonoplastes* microbial mats. Aquat Conserv 2013;23: 532–545.

[58] Suttle CA, Fuhrman JA. Enumeration of virus particles in aquatic or sediment samples by epifluorescence microscopy, In Wilhelm SW, Weinbauer MG, Suttle CA, editors. Manual of Aquatic Viral Ecology. ASLO; 2010: pp. 145–153.

[59] Prince JS, Johnson PM. Role of the digestive gland in ink production in four species of sea hares: An ultrastructural comparison. Journal of Marine Biology 2013;Article ID 209496, 5 pages. Available: 10.1155/2013/209496.

[60] Vittori M, Rozman A, Grdadolnik J, Novak U, Štrus J. Mineral Deposition in Bacteria-Filled and Bacteria-Free Calcium Bodies in the Crustacean *Hyloniscus riparius* (Isopoda: Oniscidea). PLoS ONE 2013; 8(3): e58968 10.1371/journal.pone.0058968 23554963 PMC3595210

[61] Schedl A. Log ratio methods for establishing a reference frame for chemical change. J Geol 1998;106: 211–219.

[62] Bodiguel H, Montes H, Fretigny C. Depth sensing and dissipation in tapping mode atomic force microscopy. Rev Sci Instrum 2004;75: 2529–2535.

[63] Cleveland JP, Anczykowski B, Schmid AE, Elings VB. Energy dissipation in tapping-mode atomic force microscopy. Appl Phys Lett 1998;72: 2613–2615.

[64] Mineralogy Database, Calcite mineral data. Available: http://webmineral.com/data/Calcite.shtml. Accessed 9 July 2014.

[65] Berquand A, Mazeran PE, Laval JM. Influence of volume and surface properties on phase contrast in tapping mode atomic force microscopy. Surf Sci 2003;523: 125–130.

[66] Santos F, Meyerdierks A, Pena A, Rossello-Mora R, Amann R, Antón J. Metagenomic approach to the study of halophages: the environmental halophage 1. Environ Microbiol 2007;9: 1711–1723. 17564605 10.1111/j.1462-2920.2007.01289.x

[67] Guixa-Boixareu N, Calderon-Paz JI, Heldal M, Bratbak G, Pedros-Alio C. Viral lysis and bacterivory as prokaryotic loss factors along a salinity gradient. Aquat Microb Ecol 1996;11: 215–227.

[68] Dyall-Smith M, Tang SL, Bath C. Haloarchaeal viruses: how diverse are they? Res Microbiol 2003;154: 309–313. 12798237 10.1016/S0923-2508(03)00076-7

[69] Oren A, Bratbak G, Heldal M. Occurrence of virus-like particles in the Dead Sea. Extremophiles 1997;1: 143–149. 9680320 10.1007/s007920050027

[70] Brum JR, Steward GF, Jiang SC, Jellison R. Spatial and temporal variability of prokaryotes, viruses, and viral infections of prokaryotes in an alkaline, hypersaline lake. Aquat Microb Ecol 2005;41: 247–260.

[71] Bettarel Y, Desnues A, Rochelle-Newall E. Lytic failure in cross-inoculation assays between phages and prokaryotes from three aquatic sites of contrasting salinity. FEMS Microbiol Lett 2010;311: 113–118. 10.1111/j.1574-6968.2010.02074.x 20735486

[72] Bettarel Y, Bouvier T, Bouvier C, Carré C, Desnues A, Domaizon I et al Ecological traits of planktonic viruses and prokaryotes along a full-salinity gradient. FEMS Microbiol Ecol 2001;76: 360–372. 10.1111/j.1574-6941.2011.01054.x 21255052

[73] Défarge C, Trichet J, Jaunet AM, Robert M, Tribble J, Sansone FJ. Texture of microbial sediments revealed by cryo-scanning electron microscopy. J Sediment Res 1996:66: 935–947 10.1306/D4268446-2B26-11D7-8648000102C1865D

[74] Dupraz C, Visscher P, Baumgartner LK, Reid P. Microbe-mineral interactions: early carbonate precipitation in a hypersaline lake (Eleuthera Island, Bahamas). Sedimentology 2004;51: 745–765.

[75] Glunk C, Dupraz C, Braissant O, Gallagher K, Verrecchia EP, Visscher P. Microbially mediated carbonate precipitation in a hypersaline lake, Big Pond (Eleuthera, Bahamas). Sedimentology 2010;58: 720–736. 10.1111/j.1365-3091.2010.01180.x

[76] Spadafora A, Perri E, McKenzie JA, Vasconcelos C. Microbial biomineralization process forming modern Ca:Mg carbonate stromatolites. Sedimentology 2010;57: 27–40. 10.1111/j.1365-3091.2009.01083.x

[77] Ohnesorge FM, Hörber JKH, Häberle W, Czerny C-P, Smith DPE, Binnig G. AFM review study on Pox viruses and living cells. Biophys J 1997;73: 2183–2194. 10.1016/S0006-3495(97)78250-X 9336215 PMC1181120

[78] Brum JR, Schenck RO, Sullivan MB. Global morphological analysis of marine viruses shows minimal regional variation and dominance of non-tailed viruses. ISME J 2013;7: 1738–1751. 10.1038/ismej.2013.67 23635867 PMC3749506

[79] Auguet JC, Montanie H, Lebaron P. Structure of virioplankton in the Charente Estuary (France): transmission electron microscopy versus pulsed field gel electrophoresis. Microb Ecol 2006 2;51(2):197–208. 16463133 10.1007/s00248-005-0043-0

[80] Nagasaki K, Tomaru Y, Katanozaka N, Shirai Y, Nishida K, Itakura S et al Isolation and characterization of a novel single-stranded RNA virus infecting the bloom-forming diatom *Rhizosolenia setigera* . Appl Environ Microbiol 2004;70: 704–711. 14766545 10.1128/AEM.70.2.704-711.2004 PMC348932

[81] Bettarel Y, Kan J, Wang K, Williamson KE, Cooney S, Ribblett S et al Isolation and preliminary characterisation of a small nuclear inclusion virus infecting the diatom *Chaetoceros* cf. *gracilis* . Aquat Microb Ecol 2005;40: 103–114.

[82] Eissler Y, Wang K, Chen F, Wommack E, Coats W. Ultrastructural characterization of the lytic cycle of an intranuclear virus infecting the diatom *Chaetoceros* cf. *wighamii* (bacillariophyceae) from Chesapeake Bay, USA. J Phycol 2009;45:787–797. 27034207 10.1111/j.1529-8817.2009.00705.x

[83] Tomaru Y, Takao Y, Suzuki H, Nagumo T, Nagasaki K. Isolation and characterization of a single-stranded RNA virus Infecting the bloom forming diatom *Chaetoceros socialis* . Appl Environ Microbiol 2009;75: 2375–2381. 10.1128/AEM.02580-08 19233955 PMC2675228

[84] Losic D, Rosengarten G, Mitchell JG, Voelcker NH. Pore Architecture of Diatom Frustules: Potential Nanostructured Membranes for Molecular and Particle Separations. J Nanosci Nanotechnol 2006;6: 1–8. 16736754 10.1166/jnn.2006.174

[85] Jover LF, Effler TC, Buchan A, Wilhelm SW, Weitz JS. The elemental composition of virus particles: implications for marine biogeochemical cycles. Nat Rev Microbiol 2014;12: 519–528. 10.1038/nrmicro3289 24931044

[86] Cotner JB, Hall EK, Scott JT, Heldal M. Freshwater bacteria are stoichiometrically flexible with a nutrient composition similar to seston. Front Microbiol 2010; 10.3389/fmicb.2010.00132 PMC3109486 21687767

[87] Dupraz C, Reid RP, Braissant O, Decho AW, Norman RS, Visscher P. Processes of carbonate precipitation in modern microbial mats. Earth Sci Rev 2009;96: 141–162.

[88] Goldberg M, Boskey A. Lipids and biomineralizations. Prog Histochem Cyto 1996;31: 1–187. 10.1016/s0079-6336(96)80011-8 8893307

[89] Trichet J, Défarge C. Non-biologically supported organomineralisation. Bulletin Institut Océanographique de Monaco 1995;14: 203–236. 10.1016/j.jtemb.2015.01.003 25660323

[90] Braissant O, Cailleau G, Dupraz C, Verrecchia E. Bacterially induced mineralization of calcium carbonate in terrestrial environments: the role of exopolysaccharides and amino acids. J Sediment Res 2003;73: 485–490.

[91] Chekroun KB, Rodríguez-Navarro C, González-Muñoz MT, Arias JM, Cultrone G, Rodríguez-Gallego M. Precipitation and growth morphology of calcium carbonate induced by *Myxococcus xanthus*: implication for recognition of bacterial carbonates. J Sediment Res 2004;74: 868–876. 10.1306/050504740868

[92] Bosak T, Newman DK. Microbial kinetic controls on calcite morphology in supersaturated solutions. J Sediment Res 2005;75: 190–199.

